# Complicated IVC Filter Placement in a Patient With Venous Abnormality: A Case Report

**DOI:** 10.1155/cric/8158808

**Published:** 2026-03-03

**Authors:** Maryam Mehrpooya, Faeze Salahshour, Massoud Ghasemi, Amir Mohammad Salehi

**Affiliations:** ^1^ Department of Cardiology, Imam Khomeini Medical Center, Tehran University of Medical Science, Tehran, Iran, tums.ac.ir; ^2^ Department of Radiology, Tehran University of Medical Sciences Advanced Diagnostic and Interventional Radiology Research Center, Tehran, Iran; ^3^ Department of Interventional Cardiology Research Center of Endovascular Intervention, Imam Khomeini Medical Center, Tehran University of Medical Science, Tehran, Iran, tums.ac.ir; ^4^ Fetal and Pediatric Cardiovascular Research Center, Children′s Medical Center, Tehran University of Medical Sciences, Tehran, Iran, tums.ac.ir

**Keywords:** case report, inferior vena cava, IVC filter, May–Thurner syndrome, venous anomaly

## Abstract

As a serious, prevalent, and potentially fatal condition, deep venous thrombosis (DVT) results in a huge healthcare‐related and socioeconomic burden. On the other hand, the placement of an inferior vena cava (IVC) filter is one of the last options to prevent the most dangerous complication of DVT, pulmonary embolism (PE). The patient was a 35‐year‐old man admitted to the hospital for dyspnea on exertion (NYHA class II) and lower extremity swelling. He had a history of pin implantation in his right leg and a recent history of DVT in his left calf and thigh, which was treated with warfarin. The patient was diagnosed with PE using CT angiography and was scheduled for IVC filter placement regarding the development of PE despite receiving warfarin therapy with a suitable INR. While inserting the IVC filter below the right renal vein, its legs did not fully open, and it did not go further. Thus, the procedure was terminated due to the risk of IVC perforation, and CT venography was requested, revealing the May–Thurner syndrome and a web‐like, elongated filling defect attached to the anterior IVC wall, which was an old recanalized thrombosis. Considering the anatomical disorders, we decided to retrieve the IVC filter, and the warfarin dose was increased. The present report provides a rare case of IVC anomaly management during IVC filter placement. It is recommended to look for IVC anomalies when planning interventions related to this area.

## 1. Introduction

As a serious, prevalent, and potentially fatal condition, deep venous thrombosis (DVT) is a major cause of morbidity in surgical patients, resulting in a huge healthcare‐related and socioeconomic burden [1]. The physiological conditions predisposing patients to DVT development are described as Virchow′s triad, including venous stasis, endothelial injury, and inflammation. Moreover, some of the DVT risk factors include hypercoagulable state, malignancy, pregnancy, and trauma [2, 3].

Once DVT is diagnosed, the treatment should be initiated to prevent pulmonary embolism (PE), the most dangerous complication of DVT. The first‐line treatment includes anticoagulant administration at the therapeutic level. However, the placement of an inferior vena cava (IVC) filter is indicated in patients with a contraindication of anticoagulants or recurrent PE despite receiving anticoagulative treatment at the therapeutic level [4, 5]. IVC filter complications can be classified as either acute, such as access site issues or misplacement, or as late complications. The most serious long‐term risks include migration, vena cava perforation, and filter thrombosis [6].

It is obvious that sufficient knowledge of the normal anatomy of vasculature and potential vascular anomalies is essential for the successful placement of IVC filters and the prevention of complications [7]. Herein, the present case report describes a complicated case of IVC filter placement in a patient with IVC anomaly, in which the occurrence of catastrophic events, such as IVC perforation during IVC filter placement, was prevented with the timely diagnosis.

## 2. Case Presentation

The patient is a 35‐year‐old gentleman who presented with dyspnea on exertion and lower extremity edema. His symptoms are consistent with NYHA Functional Class II, as he is comfortable at rest but experiences significant fatigue, palpitations, and shortness of breath during routine physical activities like stair‐climbing. The patient′s past surgical history is notable for an orthopedic procedure following a motor vehicle accident at 18 years of age, involving the implantation of a pin in his right leg. The hardware was electively removed 6 years postoperatively. He has never been evaluated for hypercoagulable states and reports no family history of venous thromboembolism, including deep vein thrombosis or PE.

**Figure 1:**
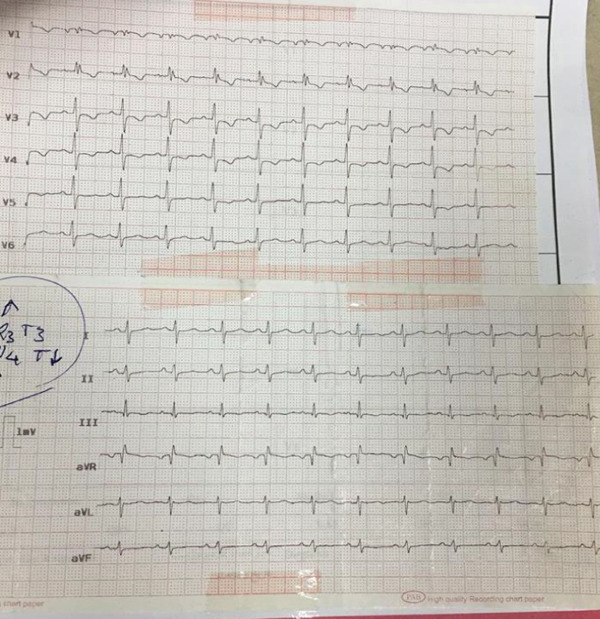
ECG was compatible with the S1Q3T3 pattern.

**Figure 2:**
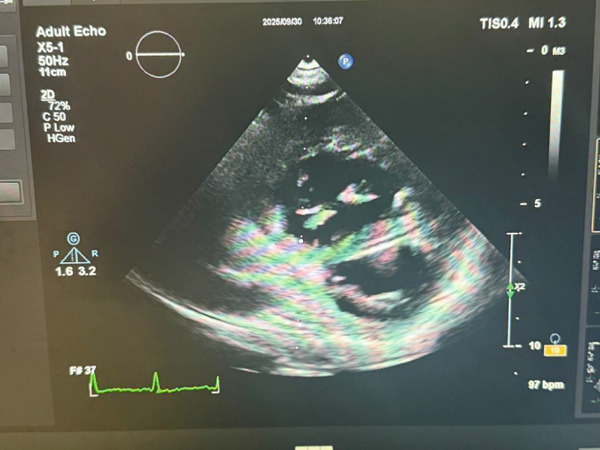
Patient′s transthoracic echocardiography finding.

Moreover, he reported a recent history of DVT (less than 1 year) in his left calf and thigh, which was treated using warfarin (5–7.5 mg daily, INR = 2.5) and had no history of smoking, hypertension, hyperlipidemia, or long‐term hospitalization. Upon examination, the patient was conscious and had a body temperature of 37°C, a heart rate of 75 bpm, a respiratory rate of 25, and a blood pressure of 100/60 mmHg, whereas the auscultation of the heart and lungs was unremarkable. The INR value at admission was 2.5. Also, he had bilateral pitting edema in his legs, which was more severe in his left leg. Thus, an ECG was ordered, revealing the S1Q3T3 pattern with sinus tachycardia, incomplete right bundle branch block, and ST depression in precordial leads (Figure 1). Moreover, the patient underwent transthoracic echocardiography, which revealed severe enlargement of the right ventricle with preserved systolic function, a D‐shaped left ventricle with an ejection fraction of 55%, a pulmonary artery pressure of approximately 45 mmHg, and no valvular abnormalities (Figure 2).

Before the procedure, a Doppler ultrasound was conducted to assess the venous system. The results showed that the right common femoral, superficial femoral, and popliteal veins were open and free of acute thrombosis. However, there was evidence of chronic, organized thrombosis in the right common and external iliac veins, which was indicated by a reduced luminal diameter. The proximal left iliac vein demonstrated a normal caliber. However, the segment coursing beneath the right iliac artery showed significant extrinsic compression without evidence of acute thrombosis. In addition, there were chronic post‐thrombotic changes in the femoral, popliteal, and anterior tibial veins, including reduced venous diameter and fully organized, nonocclusive chronic thrombus, consistent with the patient′s history of prior DVT. In the abdomen, the splenic vein, superior mesenteric vein, and portal vein were all patent and exhibited normal blood flow towards the liver.

Finally, the patients underwent computed tomography (CT) angiography, revealing filling defects in the distal part of the right main branch of the pulmonary artery, interlobar artery, and two posterior‐basal subsegmental arteries of the left lower lobe, which were compatible with PE. Thus, according to the ESC guidelines for PE [4], the patient was scheduled for IVC filter implantation due to the development of acute PE despite receiving anticoagulants at the therapeutic level.

In order to place the filter, we tried to access the IVC via the left iliac vein. However, the wire did not pass through the vein (maybe due to some previous organized thrombosis). Then, the right iliac vein was used, which was revealed to be blocked as well. Finally, we decided to use the jugular vein (File S1: https://drive.google.com/drive/folders/1oj20QpyRCBSl8ItiXRqu6kI2Cbz7EJeH?usp=drive_link). While inserting the IVC filter below the right renal vein, its legs did not fully open, and it did not go further and was tilted to one side (Figure 3) (File S2: https://drive.google.com/drive/folders/1oj20QpyRCBSl8ItiXRqu6kI2Cbz7EJeH?usp=drive_link). Using the contrast agent, a filled IVC was revealed with some narrow areas. Thus, the procedure was terminated due to the risk of IVC perforation.

Figure 3Tilted and not opened IVC filter: (a) in IVC filter placement, and (b) in CT angiography) (red arrow).

**Figure:**
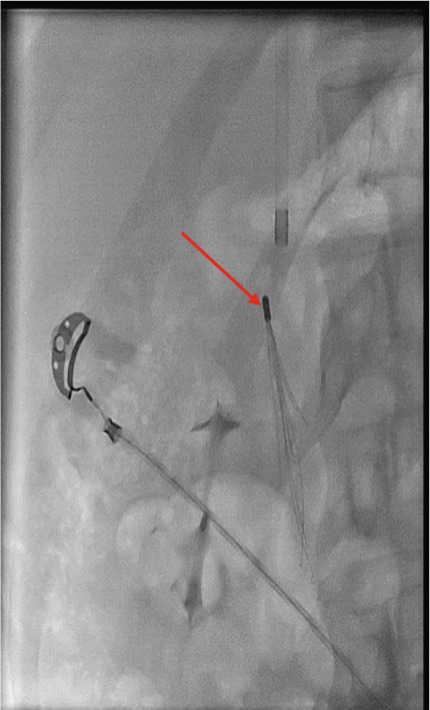
(a)

**Figure:**
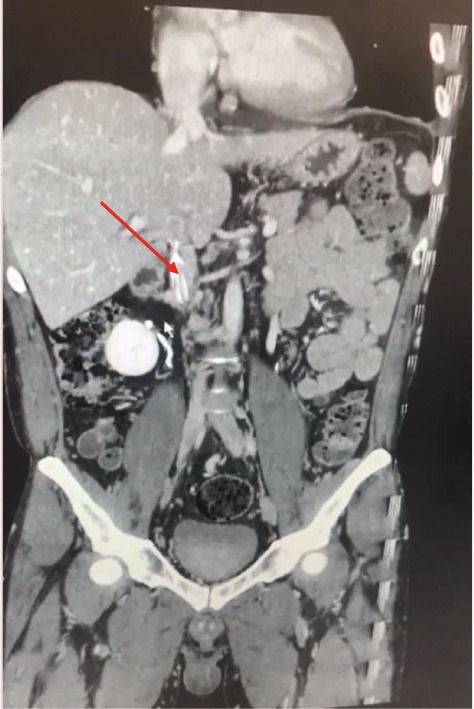
(b)

After the procedure, CT venography was requested for the patient, showing chronic thrombotic areas as significantly decreased venous diameters in the right common iliac vein and right external iliac vein. Moreover, the left common iliac vein and left external iliac vein had a normal diameter but were compressed while passing behind the right common iliac artery (May–Thurner syndrome [MTS]) (File S3: https://drive.google.com/drive/folders/1oj20QpyRCBSl8ItiXRqu6kI2Cbz7EJeH?usp=drive_link). Also, a web‐like, elongated filling defect attached to the anterior wall of the IVC was observed, revealing an old recanalized thrombosis that prevented the filter from fully opening its legs (Figure 4) (File S3: https://drive.google.com/drive/folders/1oj20QpyRCBSl8ItiXRqu6kI2Cbz7EJeH?usp=drive_link). Furthermore, a chronic thrombosis was observed in the right hepatic vein, whereas several veno–venous collaterals were seen in the right hepatic lobe. Also, the right hepatic lobe was drained by a prominent collateral vein lateral to the thrombotic hepatic vein with the same size as the hepatic veins. Finally, no evidence of Budd–Chiari syndrome was found.

Figure 4Long, longitudinal web inside the IVC (a) (red arrow) that divided the lumen into two lumens (b) (yellow arrow).

**Figure:**
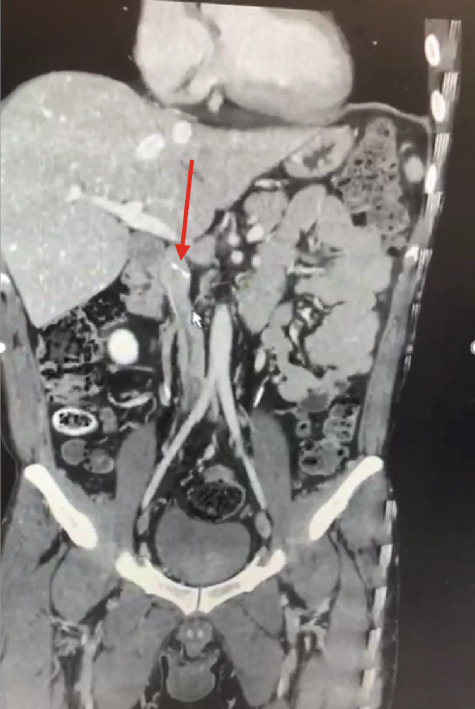
(a)

**Figure:**
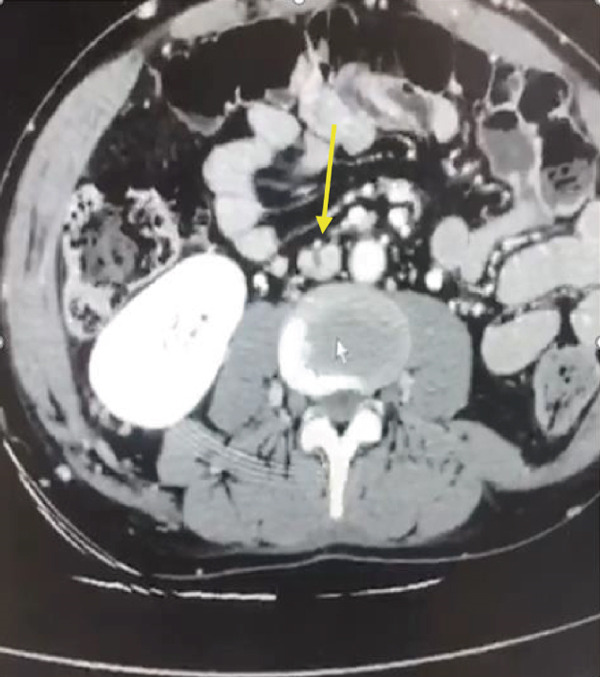
(b)

Given the identified anatomical abnormalities, the decision was made to retrieve the IVC filter. The warfarin dose was subsequently adjusted to achieve a target INR of 3 to reduce the risk of pulmonary embolism. A hematology consultation was obtained, and a comprehensive hypercoagulability evaluation was initiated. A detailed discussion was also conducted regarding further management options. Although iliac venography with potential stent placement was recommended, acknowledging the technical challenges associated with MTS, the patient declined additional interventional procedures and preferred to continue his follow‐up with a physician in his hometown.

The patient was discharged after 2 days without complications. During the 2‐year follow‐up period, he did not develop significant unilateral or bilateral lower‐extremity swelling, despite being at elevated risk for post‐thrombotic syndrome. Nevertheless, emphasis was placed on posttreatment care, including the use of compression stockings. No recurrent thromboembolic events, including DVT or PE, were observed throughout these 2 years.

## 3. Discussion

The present patient had two major venous anomalies in CT venography, including the MTS and a web‐like structure in the IVC. A long, longitudinal web inside the IVC divided the lumen into two lumens. The etiology of the condition was not known. However, it could have an embryonic origin [8, 9] or be a recanalized thrombosis in the IVC due to chronic MTS.

MTS is an anatomical variation of venous circulation caused by venous outflow obstruction due to extrinsic compression. It is secondary to partial obstruction of the left common iliac vein by an overlying right common iliac artery with subsequent entrapment of the left common iliac vein against the lumbar spine [10]. Moreover, concomitant MTS and DVT may lead to the extension of thrombus to the IVC [11]. The primary treatment for MTS has been anticoagulation. However, using only oral anticoagulants may lead to the development of recurring DVT or post‐thrombotic syndrome [12]. Therefore, a more effective treatment for MTS is an invasive endovascular procedure with stent implantation. This method should be preferred as it can also dissolve proximal thrombus [13].

As the main way of venous return from the pelvis, abdominal viscera, and lower extremities, IVC is formed from the left and right common iliac veins, which drain blood from the pelvis and lower extremities [14]. This vein receives significant tributaries from the lumbar veins, left and right renal veins, right gonadal vein, and hepatic veins as it ascends retroperitoneally at the right side of the abdominal aorta. Moreover, the azygos venous system connects to the IVC either directly or through the renal veins [15].

Diagnosing IVC disorders is challenging and can be done using magnetic resonance imaging (MRI) and CT scan. However, it is necessary to look for these uncommon vascular anomalies before attempting any surgical or radiological intervention related to the IVC, such as placing an IVC filter [16], which entails several potential complications, including IVC perforation or occlusion and filter migration, fracture, embolization (< 1%), or deployment outside of the target region. Other complications include thrombosis at the access site, insertion complications, recurrent PE, and death.

The best way to assess the IVC prior to planned filter placement is IVC venography, which is useful for determination of venous landmarks, confirmation agreements with bony vertebral levels, and exclusion of the thrombus at the intended deployment site [17]. It is usually not recommended to perform routine CT venography before IVC filter implantation, except in cases where there is suspicion of thrombosis or complications after filter implantation [18].

Thus, it is recommended to evaluate the cross‐sections of the IVC in previously taken images before any IVC filter placement; for our patient, venography (dye injection in IVC) was performed before implantation via jugular access, but considering that we only had one view, and on the other hand, because the injection was done higher than the abnormal place, therefore, we did not notice the mentioned pathology. Moreover, CT venography should be performed if no previous images are available [15] However, we did not perform the CT venography before the procedure because the condition of the patient was pretty dangerous (Spo2 was about 90% but he had dyspnea and sinus tachycardia), so he was directly referred to the Cath lab. Ideally, IVC filters are placed below the renal veins. However, in case of IVC anomaly, alternative placement locations, such as the suprarenal area, or higher doses of anticoagulants should be considered [15]. Considering the large longitudinal web in the IVC in our patient, the placement of the IVC filter was not possible in the ideal or any other places. Thus, we chose high‐dose warfarin therapy.

According to the 2020 guideline for IVC filters in the treatment of patients with venous thromboembolic disease [19], in medical centers that employ advanced techniques for filter retrieval, preprocedural imaging may not be very helpful. Instead, intraprocedural imaging techniques like venography and cone‐beam CT can be used to identify issues like filter thrombus, tilting, penetration, and fracture, which can guide therapy. However, in medical centers where advanced retrieval techniques are not available, preprocedural imaging can be useful in facilitating referrals to other medical centers with advanced techniques.

## 4. Conclusion

The present report provides a rare case of IVC anomaly management during IVC filter placement. It is recommended to look for IVC anomalies when planning interventions related to this area. The rarity of this condition and the absence of specific clinical signs make the diagnosis difficult.

**Nomenclature**
DVT: deep vein thrombosis
IVC: inferior vena cava
CT: computed tomography
MTS: May–Thurner syndrome
MRI: magnetic resonance imaging

## Author Contributions

Maryam Mehrpooya, Faeze Salahshour, and Massoud Ghasemi performed the data collection. Moreover, Amir Mohammad Salehi wrote the draft and corrected the manuscript.

## Funding

This research received no specific funding from any public, commercial, or not‐for‐profit funding agency.

## Disclosure

All authors read and approved the final manuscript for publication.

## Ethics Statement

Ethical approval to report this case was obtained from Tehran University of Medical Science. Helsinki Declaration has been followed for involving human subjects in the study. CARE guidelines were followed.

## Consent

Written informed consent was obtained from the patient for their anonymized information to be published in this article.

## Conflicts of Interest

The authors declare no conflicts of interest.

## Supporting information


**Supporting Information** Additional supporting information can be found online in sthe Supporting Information section. Videos of IVC filter placement (Videos 1 and 2) and CT venography (Video 3) https://drive.google.com/drive/folders/1oj20QpyRCBSl8ItiXRqu6kI2Cbz7EJeH?usp=drive_link.

## Data Availability

The data that support the findings of this study are available from the corresponding author upon reasonable request.
